# Telenutrition Education Is Effective for Glycemic Management in People with Type 2 Diabetes Mellitus: A Non-Inferiority Randomized Controlled Trial in Japan

**DOI:** 10.3390/nu16020268

**Published:** 2024-01-16

**Authors:** Hiroyasu Mori, Satoshi Taniguchi, Yu Tamaki, Motoyuki Tamaki, Yuko Akehi, Akio Kuroda, Munehide Matsuhisa

**Affiliations:** 1 Diabetes Therapeutics and Research Center, Institute of Advanced Medical Sciences, Tokushima University, 3-18-15 Kuramoto-cho, Tokushima 770-8503, Japan; 2 Medical IT Center, Tokushima University Hospital, 2-50-1 Kuramoto-cho, Tokushima 770-8503, Japan

**Keywords:** telenutrition, nutrition education, glycemic management, type 2 diabetes mellitus, non-inferiority trial

## Abstract

This study examined the non-inferior efficacy of telenutrition education compared with face-to-face nutrition education in managing glycemic control in people with type 2 diabetes mellitus (T2DM). Participants had T2DM and a glycated hemoglobin (HbA1c) ranged 6.5–9.5%. Thirty participants were randomly assigned to either the telenutrition or face-to-face nutrition education group. During the 32-week intervention period, the participants received four sessions on nutrition education from a registered dietitian at the hospital. The telenutrition group received remote education via a videoconferencing platform. Face-to-face nutrition education was conducted using paper-based instructions. The main outcome measure was the non-inferiority of HbA1c levels in the telenutrition group compared to the face-to-face nutrition group. The non-inferiority of telenutrition education was considered valid if the intergroup difference in the mean values of the change in HbA1c had a bilateral 95% confidence interval (CI) upper limit below 0.40%. The intergroup difference in the mean HbA1c change from baseline to the fourth nutrition education session was −0.11 (95% CI −0.54–0.32) for both groups. The upper limit of the bilateral 95% CI was 0.32%, which was below the 0.40% non-inferiority margin (non-inferiority test; *p* = 0.011). Telenutrition education was not inferior to face-to-face nutrition education for glycemic management in people with T2DM.

## 1. Introduction

Nutrition education is an important instructional tool for glycemic and weight management in people with type 2 diabetes mellitus (T2DM) [1]. Face-to-face nutrition education for people with T2DM is beneficial for glycemic management and weight loss [2,3]. The use of telenutrition technology offers various advantages, especially for routine care and in cases where services do not require direct interaction with the patient [4,5]. Moreover, telenutrition education can be more time- and cost-efficient than other educational methods [6,7,8].

Dietitians are advised to choose a remote nutritional guidance when face-to-face guidance is necessary but not difficult. However, the efficacy of remote nutrition education for individuals with T2DM is unclear, and it is necessary to evaluate its non-inferiority to face-to-face nutrition education through randomized controlled trials (RCTs). Therefore, the benefits of telenutrition education for glycemic management need to be identified in RCTs [9]. Therefore, this RCT aimed to evaluate the non-inferior efficacy of telenutrition education compared with face-to-face nutrition education for glycemic management among people with T2DM.

## 2. Materials and Methods

### 2.1. Study Design

This intervention study employed a non-inferiority randomized controlled intervention design. The physicians and medical staff who collected the outcomes were blinded to group allocation, while the single registered dietitian who provided nutrition education was not blinded to group allocation. This study was approved by the Ethics Committee of the University of Tokushima (No. 2378-3, corresponding approval date: 31 August 2016) and registered in the University Hospital Medical Information Network (No. 000018295). Written informed consent for participation and publication of this study was obtained from all participants.

### 2.2. Participants

The study was conducted at a single site at Tokushima University Hospital, Tokushima, Japan. The participants were recruited between February and March 2017, and the interventions were conducted from March to November 2017. The inclusion criteria for participants were as follows: a diagnosis of T2DM and a glycated hemoglobin (HbA1c) value from 6.5% to 9.5%. The exclusion criteria were as follows: a worsening HbA1c level of ≥1% among those with HbA1c values between 6.5% and 9.5% in the past three months, serious cardiovascular disease, advanced kidney injury, or active retinopathy. Additionally, participants who had received nutrition education over the past two years were not included. Thirty-five participants expressed interest in the study. Of these, 30 participants who were eligible for participation signed the consent form and were enrolled in this study. After the baseline assessment, the research staff randomly assigned eligible participants to one of two groups in a 1:1 ratio according to the randomization list order.

### 2.3. Nutrition Education Program

A registered dietitian provided nutrition education for glycemic management according to the instructions of the physicians. Physicians recommended diet therapy for registered dietitians based on the Japanese Clinical Practice Guideline for Diabetes 2016–2017 (covering total energy, as well as the intake of carbohydrates, fats, proteins, and salt), [10] and the Food Exchange Lists Dietary Guidance for Persons with Diabetes (covering the intake of each food group: cereals, potatoes, fish and shellfish, meat, eggs, milk and dairy products, bean and soybean products, vegetables, fruits, fat and oil, sugar, and confectioneries) from the Japan Diabetes Society [11]. Additionally, counseling materials for nutrition education considering stage of behavior change of T2DM individuals were chosen in accordance with the Food and Nutrition Education Manual, prepared by the Japan Dietetic Association [12]. During the 32-week intervention period, the participants received four sessions on nutrition education from a registered dietitian at the hospital. All participants visited the hospital for medical care at eight-week intervals. The registered dietitian provided an individualized nutrition education plan based on the Nutrition Care Process Model (e.g., the four steps of nutrition assessment, diagnosis, intervention, and monitoring and evaluation) during telenutrition and face-to-face sessions [13]. At baseline, the registered dietitian and participants set SMART (i.e., specific, measurable, achievable, relevant, and time-bound) goals for diet therapy with optimal glycemic management as the outcome [13]. During the intervention period, participants were asked to maintain the same amount of physical activity as before the intervention. During the intervention period, each group underwent four face-to-face medical examinations with a diabetologist.

### 2.4. Telenutrition Education Materials

**Figure 1:**
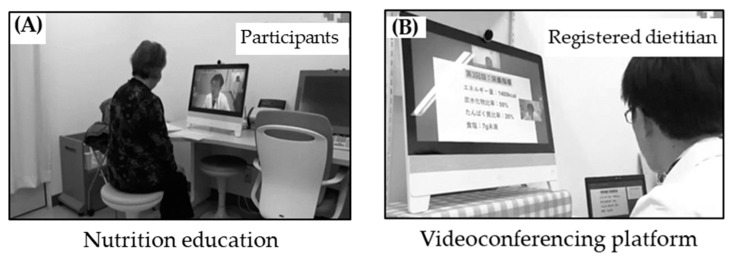
Live action of telenutrition education. The telenutrition education was provided via a Health Insurance Portability and Accountability Act-compliant videoconferencing platform (Cisco Systems, Inc., DX 80, USA); participants used a webcam that was provided to them by the research team. A LAN cable connection was formed between the registered dietitian’s personal computer and the videoconferencing platform. The registered dietitian activated the videoconferencing platform from the consulting room to start the telenutrition education sessions. All educational materials were provided electronically and were to be opened using Microsoft PowerPoint, Microsoft Excel, Microsoft Word, and Adobe Acrobat. Consulting room (**A**): Participants in the telenutrition education group. Consulting room (**B**): Registered dietitian providing telenutrition education.

Telenutrition education was delivered via a Health Insurance Portability and Accountability Act-compliant videoconferencing platform (Cisco Systems, Inc., DX 80, San Jose, CA, USA. IIJ Global Solutions Inc., Tokyo, Japan). Participants used a webcam provided to them by the research team (Figure 1). The dietitian was skilled in using the videoconferencing platforms. Telenutrition education was provided via a LAN cable connection between the registered dietitian’s personal computer and a videoconferencing platform. The registered dietitian activated the videoconferencing platform from the consulting room to begin the telenutrition education session. Telenutrition education materials were provided electronically (to be opened using Microsoft PowerPoint, Microsoft Excel, Microsoft Word, and Adobe). Prior to the telenutrition education sessions, the medical staff admitted the participants to another consulting room and activated a videoconferencing platform. The registered dietitian provided telenutrition education via the videoconferencing platform, asking for clinical information on HbA1c and self-monitoring information such as blood glucose, weight, blood pressure, and dietary records from both the medical staff and participants. The registered dietitian could also use a touch pen to write instructional comments and present them on the participant’s display. Before remote nutrition education, security risks, countermeasures, and responsibilities were explained to the participants and were agreed upon. The operating system and relevant PC software were updated or installed, as necessary. When starting up the videoconferencing platform, a special ID and password were used and were kept secret only from the registered dietitian and medical staff. The registered dietitian conducting the remote nutrition education was provided with information on the security risks of the procedure through medical information training and other means as appropriate.

Various communication techniques were implemented during telenutrition education sessions. For instance, participants were instructed to greet each other and introduce themselves at the start of the telenutrition sessions to put them at ease and confirm that it was the participant who was in front of the display.

The dietitian spoke slowly, clearly, and in plain language, repeating important points of nutrition therapy several times to ensure that the participants understood them. The dietitian used nonverbal communication such as gestures, facial expressions, and nodding. In addition to verbal communication, nonverbal information, such as the patient’s facial expressions, emotions, and movements, was also considered important. The dietitian also used open-ended questions that allowed the participants to converse freely. At the end of the session, the dietitian summarized the session and asked the participants questions to determine whether there were any issues.

### 2.5. Face-to-Face Nutrition Education Materials

Face-to-face nutrition education materials were provided to the individuals via paper-based instruction, such as pamphlets and textbooks. The registered dietitian obtained participants’ clinical information (including HbA1c, plasma glucose, body weight, blood pressure, and dietary records) in person from both the medical staff and participants.

### 2.6. Outcome Measures

HbA1c level was the primary outcome. The secondary outcomes were body weight, systolic blood pressure (SBP), diastolic blood pressure (DBP), stage of behavior change, adherence to diet therapy, total energy, macronutrients, and salt intake.

Each outcome was measured at baseline (February to March 2017), during the intervention (after session 1: April to May 2017, 2: June to July 2017, and 3: August to September 2017), and at the end of the intervention (after session 4: October to November 2017) by blinded medical staff (Figure S1).

### 2.7. Clinical Date

Age, sex, duration of T2DM, HbA1c level, estimated glomerular filtration rate, diabetic neuropathy, diabetic retinopathy (including simple diabetic retinopathy, pre-proliferative diabetic retinopathy, and proliferative diabetic retinopathy), hypertension, dyslipidemia, SBP, DBP, anti-diabetic medications (insulin therapy, sulfonylureas, metformin, alpha-glucosidase inhibitors, glinides, thiazolidinedione, dipeptidyl peptidase-4 inhibitors, sodium-dependent glucose transporter 2 inhibitors, glucagon-like peptide-1 receptor agonists, and statins renin-angiotensin system inhibitors), and physician-prescribed medicines were collected from the participants’ medical records [10].

### 2.8. Assessment of Physical Activity

Physical activity was assessed using the International Physical Activity Questionnaire as a metabolic equivalent task per week [14].

#### Assessment of Body Composition

Body weight and BMI were measured using a multifrequency bioelectrical impedance analyzer (In Body Japan, Tokyo, Japan).

### 2.9. Assessments of Health Behavior Change

Based on Prochaska’s transtheoretical model of health behavior change, medical staff members were interviewed regarding the five behavior change stages of adherence to diet therapy (pre-intention stage = 1–2, intention stage = 3–4, preparation stage = 5–6, action stage = 7–8, and maintenance phase = 9–10) [15,16].

### 2.10. Assessment of Total Energy, Macronutrients, and Salt Intake

At baseline, all participants were provided with a digital scale (TANITA, KJ-110M) to weigh food items, and were asked to record the amount of food consumed at each meal. In case of missing responses, individual interviews were conducted with participants regarding their food intake status. The total energy, macronutrient, and salt intake survey included a food weighing method in which food intake was documented for seven consecutive days after sessions 0, 1, 2, 3, and 4. Energy, macronutrients, and salt intake were calculated using the Fifth Revised Edition of the Standard Tables of Food Composition in Japan and the 2020 Japanese Dietary Reference Intakes using nutrition calculation software (version 9.0; Excel Eiyou, Kenpakusha, Tokyo, Japan, release date February 2020) [17].

### 2.11. Statistical Analysis

All statistical analyses were performed using R (version 4.3.1) and SPSS (version 25.0 (SPSS, IBM, Japan). All outcome data were checked for normality using the Shapiro–Wilk test and are shown as the mean ± standard deviation or median [25th and 75th percentiles]. Participants who received nutrition education at baseline were included in the statistical analysis.

A sample size of 24 participants (12 participants for each group) was estimated to yield an α level of 0.05, and a power of 80% for the upper confidence limit of the mean difference in change in HbA1c between both groups not exceeding 0.40%, assuming a standard deviation of 0.35% for a true difference of 0.0%.

Between-group differences in the distribution of baseline clinical characteristics and outcome data were analyzed using an unpaired *t*-test, Mann–Whitney U test, or chi-squared test. Within-group differences in primary and secondary outcome data (HbA1c, body weight, SBP, DBP, behavior change stage, total energy, and carbohydrate, protein, fat, and salt intake) at baseline, compared to during or at the end of the intervention, were analyzed using a repeated measure one-way analysis of variance or Friedman’s test. A two-sided *p*-value of <0.05 indicated significance in the following analyses: unpaired *t*-test, Mann–Whitney U test, chi-squared test, repeated measure one-way analysis of variance, and Friedman’s test.

A non-inferiority trial seeks to determine whether a new treatment is worse than a standard treatment by more than an acceptable amount. Statistically, it is performed by rejecting the one-sided null hypothesis that one intervention is appreciably worse than another. Consequently, the margin of non-inferiority is defined as the treatment effect on the primary patient outcome. This margin is predetermined by a subject matter expert with specific knowledge of the differences between new and standard treatments. The non-inferiority of telenutrition education (in comparison to face-to-face nutrition education) was deemed valid if the upper limit of the bilateral 95% confidence interval (CI) of the intergroup difference in mean values of the change in HbA1c fell below 0.40% [18,19,20]. The non-inferiority test result was significant (one-sided *p* < 0.025).

## 3. Results

**Figure 2:**
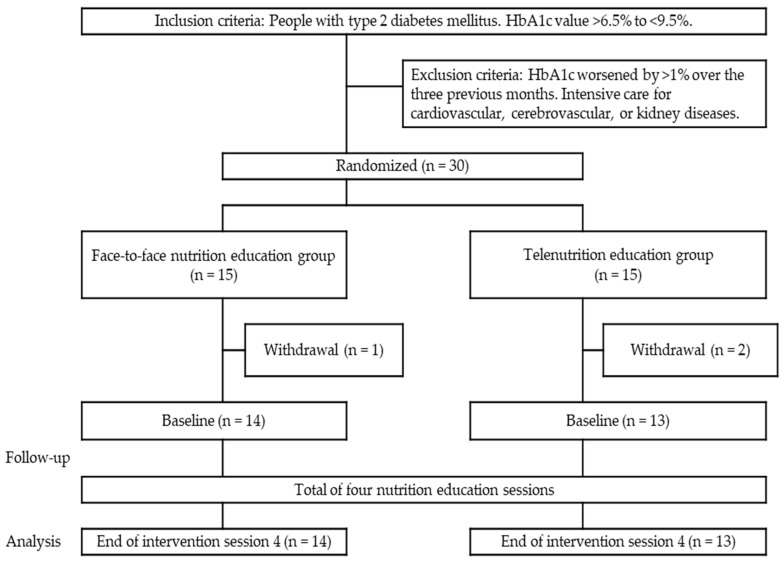
Flowchart showing the participants with type 2 diabetes. After random assignment to the two groups, one participant in the face-to-face nutrition education group and two in the telenutrition education group withdrew before the first nutrition education session. Consequently, 14 and 13 participants in the face-to-face and telenutrition education groups, respectively, were included in the final analysis. Abbreviations: HbA1c, glycated hemoglobin.

After random assignment to the two groups, one participant in the face-to-face nutrition education group and two in the telenutrition education group withdrew before the first nutrition education session. Consequently, 14 and 13 participants in the face-to-face and telenutrition education groups, respectively, were included in the final analysis (Figure 2). No serious adverse events (e.g., severe hypoglycemia, hospitalization, or leakage of medical information) occurred during the intervention period. At baseline, 10 participants in the telenutrition education group and 11 in the face-to-face nutrition education group required education on total energy intake management. Additionally, 10 participants in each group required education on carbohydrate intake management.

The baseline clinical characteristics of participants with T2DM in the face-to-face and telenutrition education groups are presented in Table 1 and Table S1. There were no statistically significant differences in the clinical characteristics or primary/secondary outcomes between the groups at baseline.

**Figure 3:**
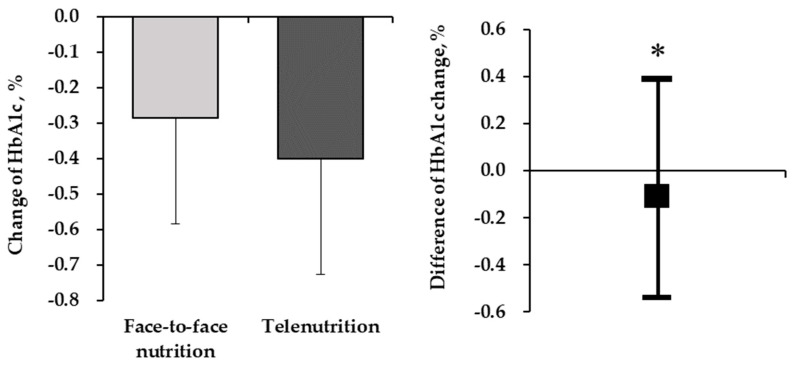
The intergroup difference in the mean change in HbA1c, from the beginning of the nutrition education sessions until after the fourth session for both groups. HbA1c change from baseline to the end of intervention for each group. Data are shown as mean ± standard deviation. Differences in HbA1c levels between groups. Data are shown as the mean and 95% confidence interval. Non-inferiority test; * *p* = 0.011.

The primary and secondary outcomes at the end of the intervention in the face-to-face and telenutrition education groups are presented in Table 2 and Table S2, and Figure S2. After session 4, both the face-to-face and telenutrition education groups showed a significant mean decrease in HbA1c, body weight, SBP, total energy, carbohydrate, and salt intake amount (*p* < 0.05 for both groups). Furthermore, both groups showed a significant mean increase in the behavior change stage score (*p* < 0.05 for both groups). The intergroup difference in the mean change in HbA1c, from the beginning of the nutrition education sessions until after the fourth session, was −0.11 for both groups (95% CI, −0.54 to 0.32; Figure 3). The upper limit of the bilateral 95% CI was 0.32%, which was well below the 0.40% non-inferiority margin (non-inferiority test; *p* = 0.011).

The primary and secondary outcomes during the intervention in the face-to-face and telenutrition education groups are presented in Table S3 and Figure S2. After session 3, both the face-to-face and telenutrition education groups showed a decrease in HbA1c, body weight, total energy, carbohydrate, and salt intake (*p* < 0.05 for both groups), and an increase in the behavior change stage score (*p* < 0.05 for both groups).

There were no significant intergroup differences regarding the medicines used for diabetes at baseline, during or at the end of the intervention.

## 4. Discussion

This study showed that HbA1c levels significantly improved in both the face-to-face and telenutrition education groups at the end of the intervention. Moreover, telenutrition education was not inferior to face-to-face nutrition education with respect to the HbA1c reduction in people with T2DM.

A previous study reported the efficacy of face-to-face nutrition education for glycemic management in people with T2DM [21]. Although the efficacy of telenutrition education (e.g., via text messages and telephone coaching) for glycemic management in people with T2DM has been reported [22], few studies have examined the efficacy of telenutrition education techniques using information and communication technology (e.g., videoconferencing via the internet) [23]. In nutrition education, communication based on audio and visual information is important for gathering lifestyle and dietary information from the individuals. Telephone-based nutrition education is limited because it only involves communication through audio information using a microphone and speaker. In a previous study [24], telephone-based telenutrition education did not improve dietary self-management, such as salt intake. In contrast, in this study, videoconferencing-based telenutrition education improved energy and salt intake in people with T2DM. Therefore, voice-only telenutrition education may have a limited instructional efficacy [24]. Conversely, the efficacy of videoconferencing-based nutrition education is greater because it uses a microphone, speakers, video camera, and display to deliver both auditory and visual information. There were no problems with participants regarding audio, visual, or auditory information during the telenutrition education sessions. An earlier systematic review found that healthcare providers can assist people in using information and communication technology, thereby improving patient adherence to treatment [22].

Telenutrition is an innovative approach that enables the delivery of healthcare services in remote areas through the use of digital devices, the internet, software, and telecommunication networks [9,22,23]. The digitization of healthcare using telenutrition and other new digital approaches has evolved over the last few years, and the unique circumstances of the coronavirus disease 2019 (COVID-19) pandemic have led to its rapid and widespread adoption [4,5]. This study reported a method of nutrition education using a videoconferencing platform, and showed that telenutrition education can contribute to the treatment of diabetes for glycemic management by a registered dietitian.

The efficacy of text messages and telephone coaching in reducing HbA1c and fasting blood glucose levels in people with T2DM has been reported [25]. Furthermore, in comparison to usual care, telenutrition education using a videoconferencing system for 12 weeks increased the healthy food intake and significantly reduced body weight in obese men [23]. Another study reported that a six-month video-based lifestyle education program significantly reduced HbA1c levels and body weight in people with T2DM [26]. Although the abovementioned studies were not randomized intervention studies that determined the non-inferiority of telenutrition education when compared with face-to-face nutrition education, they demonstrated that telenutrition education is a teaching method that can be used for therapeutic purposes such as blood glucose management. The current results demonstrated that telenutrition education led to a reduction in participants’ HbA1c levels by −0.40 (0.1) % (the mean and standard deviation, % change in HbA1c). Previous studies have used a 0.3–0.4% reduction in HbA1c as a measure to assess the efficacy of medication and glucose monitoring devices for blood glucose management [27,28]. Therefore, telenutrition education can be effective for people with T2DM in this study as well.

The participants’ stages of behavior change in relation to diet therapy significantly improved not only from face-to-face nutrition education (the median, 25th and 75th percentiles of behavior change stage, baseline; 4 [3, 5], session 4; 7 [5, 8]) but also from telenutrition education (the median, 25th and 75th percentiles of behavior change stage, baseline; 4 [3, 4], session 4; 7 [5, 7]), thereby significantly decreasing body weight, total energy, and carbohydrate intake. Few studies have measured individuals’ behavioral change stage, body weight, total energy, and carbohydrate intake; therefore, the results of this study provide valuable clinical data. At the end of the intervention, the telenutrition education group lost 3 (1) % of their body weight (the mean and standard deviation, % change in body weight). The weight loss observed in this study supports the efficacy of telenutrition education. Weight loss of 1–3% per individual significantly improves LDL and HDL cholesterol, triglycerides, and HbA1c; further, weight loss of 3–5% per individual significantly improves blood pressure and fasting blood glucose [29]. This study showed a decrease in participants’ HbA1c levels and body weight, suggesting that dietary management was achieved through improved behavioral changes associated with the telenutrition education intervention, leading to better glycemic management.

Interestingly, the participants in the telenutrition and face-to-face nutrition education groups showed similar behavioral improvement. The participants’ behavior changed from “interested in the diet (intention stage) and ready (preparation stage) to implement it (action stage).” These findings imply that telenutrition education is capable of ensuring adherence to and motivation toward the diet, thereby improving glycemic management [30]. Conversely, people with low motivation (pre-intention stage) during their health behavior change have difficulty adhering to diet therapy [31,32]. Therefore, telenutrition education on glycemic management could positively impact people with T2DM whose health behavior changes correspond to the intention and preparation stages.

This randomized controlled study is associated with several limitations. First, it was conducted at a single center in Japan. Second, the sample size of this study was relatively small. As a result, the results of this study should be considered as a preliminary report, while future intervention studies should be conducted at multiple centers and include larger samples. In addition, this study should have been conducted on a diverse group of participants, including those who are younger (average age less than 60 years), professionally active, and whose lifestyles significantly influence their eating habits. However, due to the small sample size in this study, sub-analyses based on age and lifestyle differences could not be conducted. Future studies should therefore compare the advantages and disadvantages of the educational benefits of telenutrition and face-to-face nutrition education by age and lifestyle through a randomized controlled trial with a larger sample size. Third, food intake was self-reported by the participants; thus, there may be a potential for bias [33,34]. Fourth, we did not conduct a satisfaction survey based on the participant responses. Future studies should compare the advantages and disadvantages of the educational benefits of telenutrition and face-to-face nutrition education through RCT. Fifth, the study design lacked a control group of participants who did not receive nutritional education. Therefore, future studies should include a control group to confirm the effects of telenutrition education observed in this study. Finally, the mean HbA1c level was <8% in both the telenutrition and face-to-face nutrition education groups, suggesting that intervention studies should also be conducted in people with poorly controlled T2DM (mean HbA1c >8%).

## 5. Conclusions

In this non-inferiority RCT, participants with T2DM were randomly assigned to receive either telenutrition or face-to-face nutrition education. Telenutrition education was not inferior to face-to-face nutrition education in glycemic management among people with T2DM.

## Figures and Tables

**Table 1:** Baseline clinical characteristics of the 27 participants in the face-to-face nutrition and telenutrition education groups.

Baseline of Intervention: Before Session 1	Face-to-Face Nutrition Group (n = 14)	Telenutrition Group (n = 13)	*p* vs. between Groups ^1^
Age, years	64.0 ± 9.2	62.8 ± 10.7	0.766
Sex, Female/male	10/4	9/4	0.999
Duration of type 2 diabetes, year	14.3 ± 8.2	16.9 ± 11.1	0.488
HbA1c, %	7.1 ± 1.0	7.7 ± 1.2	0.166
Body weight, kg	66.6 ± 9.0	66.9 ± 10.2	0.950
BMI, kg/m^2^	27.6 ± 3.2	27.5 ± 4.6	0.958
SBP, mmHg	133 ± 13	133 ± 7	0.930
Behavior change stage, score	4 [3, 5]	4 [3, 4]	0.430
Total energy intake, kcal/day	1964 ± 295	1944 ± 354	0.874

Data are shown as the mean ± standard deviation or median [25th and 75th percentiles]. Abbreviations: HbA1c, glycated hemoglobin; BMI, body mass index; SBP, systolic blood pressure. ^1^ Comparison between groups: unpaired *t*-test, Mann–Whitney U test, or chi-squared test.

**Table 2:** Primary and secondary outcomes at the end of intervention in 27 participants in the face-to-face nutrition and telenutrition education groups.

End of Intervention: After Session 4	Face-to-Face Nutrition Group (n = 14)	^1^ *p* vs. Baseline	Telenutrition Group (n = 13)	^1^ *p* vs. Baseline
Primary outcome				
HbA1c, %	6.8 ± 0.8	0.002	7.3 ± 1.1	0.012
Secondary outcome				
Body weight, kg	65.7 ± 10.8	0.019	64.9 ± 8.6	0.008
SBP, mmHg	130 ± 13	0.016	130 ± 13	0.025
Behavior change stage, score	7 [5, 8]	0.001	7 [5, 7]	0.001
Total energy intake, kcal/day	1841 ± 298	0.001	1847 ± 321	0.001

Data are shown as the mean ± standard deviation or median [25th and 75th percentiles]. Abbreviations: HbA1c, glycated hemoglobin; SBP, systolic blood pressure. ^1^ Comparison within groups: repeated measure one-way analysis of variance or Friedman’s test.

## Data Availability

All clinical outcomes are available from the corresponding author upon reasonable request.

## Supplementary Materials

The following supporting information can be downloaded from https://www.mdpi.com/article/10.3390/nu16020268/s1, Figure S1: Collection of primary and secondary outcomes during the intervention period. Table S1: Baseline clinical characteristics of the 27 participants in the face-to-face nutrition and telenutrition education groups; Table S2: Secondary outcomes during intervention in the 27 participants in the face-to-face nutrition and telenutrition education groups; Table S3: Primary and secondary outcomes during intervention in 27 participants in the face-to-face nutrition and telenutrition education groups. Figure S2. HbA1c change from baseline to the end of intervention for each group. CONSORT 2010 checklist of information to include when reporting a randomized trial.
