# Supplementary material for: Telenutrition Education Is Effective for Glycemic Management in People with Type 2 Diabetes Mellitus: A Non-Inferiority Randomized Controlled Trial in Japan
Source: Nutrients. 2024 Jan 16;16(2):268. doi: 10.3390/nu16020268 (PMC10819819; doi:10.3390/nu16020268)
Supplement: Supplementary file 1 [file nutrients-16-00268-s001.zip › nutrients-2808477-supplementary.pdf]

Figure S1. Collection of primary and secondary outcomes during the intervention period

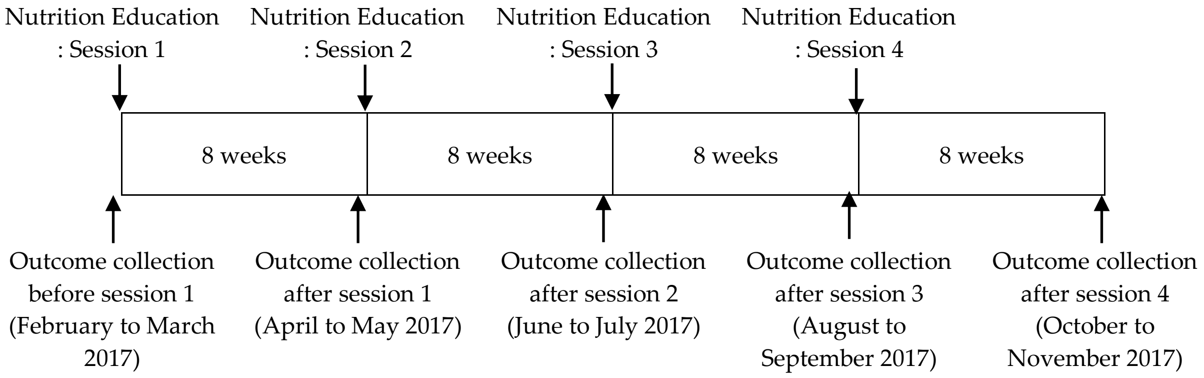

Table S1. Baseline clinical characteristics of the 27 participants in the face-to-face nutrition and telenutrition education groups

Baseline of intervention: Before session 1	Face-to-face nutrition group (n = 14)	Telenutrition group (n = 13)	[1] *P* vs. between groups
DBP, mmHg	82±9	84±10	.510
eGFR, mL/min/1.73 m^2	76±22	70±19	.503
eGFR < 30 mL/min/1.73 m^2, n	3	1	.596
Diabetic neuropathy, n	8	9	.695
Diabetic retinopathy, SDR/PPDR/PDR, n	2/1/0	1/1/0	.862
Hypertension, n	8	7	.863
Dyslipidemia, n	7	7	.842
Physical activity, MET min/week	759 [323, 1386]	693 [462, 1386]	.616
Therapy of insulin, n	8	11	.209

Sulfonylureas, n	0	1	.481
Metformin, n	6	5	.816
Alpha-Gls, n	3	3	.999
Glinides, n	1	2	.596
TZDs, n	2	0	.481
DPP-4 inhibitors, n	7	5	.547
SGLT2 inhibitors, n	2	3	.648
GLP1-RAs, n	5	6	.581
Statin, n	7	7	.842
RAS inhibitors, n	7	6	.842
Total protein intake, g/day	74±15	76±11	.658
Total protein energy ratio, g/kcal	15.0±2.5	15.7±1.3	.383
Total fat intake, g/day	55±5	55±2	.808
Total fat energy ratio, g/kcal	30.1±3.1	29.4±2.8	.539
Total carbohydrate intake, g/day	262±45	263±42	.812
Total carbohydrate energy ratio, g/kcal	54.8±5.2	54.9±2.1	.990
Total salt intake, g/day	10.5±1.6	10.8±2.7	.737
Physician prescribed total energy intake, kcal/day	1,871±210	1,857±141	.845
Physician prescribed total protein energy ratio, %	16.3±4.1	16.2±2.9	.924
Physician prescribed total fat energy ratio, %	27.3±4.1	26.2±2.9	.432
Physician prescribed total carbohydrate energy	56.4±2.2	57.7±2.5	.196

ratio, %			
Physician prescribed total salt intake, g/day	7±1	7±1	.905

Data are shown as the mean ± standard deviation, or median [25th and 75th percentiles].

Abbreviations: DBP, diastolic blood pressure; eGFR, estimated glomerular filtration rate; SDR, simple diabetic retinopathy; PPDR, pre-proliferative diabetic retinopathy; PDR, proliferative diabetic retinopathy; MET, metabolic equivalent task; Alpha-Gls, alpha glucosidase inhibitors; TZDs, thiazolidinedione; DPP-4, dipeptidyl peptidase-4; SGLT2, sodium-dependent glucose transporter 2; GLP-1 RAs, glucagon-like peptide-1 receptor agonists; RAS, renin-angiotensin system.

[1]Comparison between groups: unpaired t-test, Mann–Whitney U test, or chi-squared tests.

Table S2. Secondary outcomes during intervention in the 27 participants in the face-to-face nutrition and telenutrition education groups

End of intervention: After session 4	Face-to-face nutrition (n = 14)	[1] *P* vs. baseline	Telenutrition (n = 13)	[1] *P* vs. baseline
Secondary outcome				
DBP, mmHg	80±7	.437	82±9	.382
Total protein intake, g/day	68±15	.012	72±10	.024
Total protein energy ratio, g/kcal	14.7±2.6	.634	15.7±1.9	.999
Total fat intake, g/day	63±12	.016	60±15	.001
Total fat energy ratio, g/kcal	30.4±3.2	.944	29.1±2.8	.786

Total carbohydrate intake, g/day	251±49	.006	254±42	.020
Total carbohydrate energy ratio, g/kcal	54.9±5.6	.998	55.1±2.2	.926
Total salt intake, g/day	9.5±1.5	.001	9.7±2.3	.001

Data are shown as the mean ± standard deviation or median [25th and 75th percentiles].

Abbreviations: DBP, diastolic blood pressure.

[1] Comparison within groups: repeated measure one-way analysis of variance or Friedman's test.

Table S3. Primary and secondary outcomes during intervention in 27 participants in the face-to-face nutrition and telenutrition education groups

During intervention: After session 1	Face-to-face nutrition (n = 14)	[1] *P* vs. baseline	Telenutrition (n = 13)	[1] *P* vs. baseline
Primary outcome				
HbA1c, %	7.0±1.1	.202	7.4±1.0	.093
Secondary outcome				
Body weight, kg	66.7±10.6	.979	65.6±8.9	.150
SBP, mmHg	132±14	.624	130±8	.132
DBP, mmHg	81±7	.950	82±10	.778
Behavior change stage, score	4 [4, 5]	.403	4 [3, 4]	.999
Total energy intake, kcal/day	1,933±255	.905	1,923±336	.683

Total protein intake, g/day	72±14	.735	75±12	.991
Total protein energy ratio, g/kcal	14.5±2.4	.275	15.7±1.3	.999
Total fat intake, g/day	66±11	.858	63±15	.662
Total fat energy ratio, g/kcal	30.2±2.8	.999	29.4±2.5	.990
Total carbohydrate intake, g/day	262±45	.713	263±42	.918
Total carbohydrate energy ratio, g/kcal	55.3±4.6	.751	54.9±1.9	.990
Total salt intake, g/day	10.4±1.6	.929	10.7±2.8	.994
During intervention: After session 2	Face-to-face nutrition (n = 14)	[1] *P* vs. baseline	Telenutrition (n = 13)	[1] *P* vs. baseline
Primary outcome				
HbA1c, %	7.0±1.1	.140	7.5±1.0	.119
Secondary outcome				
Body weight, kg	66.0±10.4	.089	65.0±8.7	.012
SBP, mmHg	131±14	.277	131±14	.147
DBP, mmHg	81±8	.965	81±9	.208
Behavior change stage, score	5 [4, 5]	.151	4 [3, 4]	.809
Total energy intake, kcal/day	1,897±292	.026	1,919±337	.532
Total protein intake, g/day	71±16	.451	74±10	.303
Total protein energy ratio, g/kcal	15.1±2.7	.999	15.5±1.5	.941

Total fat intake, g/day	63±11	.087	65±15	.604
Total fat energy ratio, g/kcal	30.2±2.8	.999	30.4±2.2	.109
Total carbohydrate intake, g/day	260±45	.062	259±44	.297
Total carbohydrate energy ratio, g/kcal	54.7±4.8	.998	54.1±1.5	.240
Total salt intake, g/day	10.2±1.7	.200	10.4±2.6	.259
During intervention: After session 3	Face-to-face nutrition (n = 14)	[1] *P* vs. baseline	Telenutrition (n = 13)	[1] *P* vs. baseline
Primary outcome				
HbA1c, %	6.9±0.8	.012	7.3±1.0	.014
Secondary outcome				
Body weight, kg	65.8±10.8	.027	65.0±8.7	.013
SBP, mmHg	130±13	.078	130±13	.050
DBP, mmHg	80±7	.527	82±8	.345
Behavior change stage, score	5 [4, 7]	.013	5 [4, 6]	.029
Total energy intake, kcal/day	1,857±299	.002	1,862±315	.010
Total protein intake, g/day	68±16	.013	73±10	.203
Total protein energy ratio, g/kcal	14.6±2.7	.404	15.8±1.2	.989
Total fat intake, g/day	63±10	.045	62±15	.056
Total fat energy ratio, g/kcal	30.5±2.6	.834	29.7±2.4	.772
Total carbohydrate intake,	254±47	.018	253±40	.008

g/day				
Total carbohydrate energy ratio, g/kcal	54.9±4.8	.999	54.4±1.9	.748
Total salt intake, g/day	9.8±1.4	.002	10.0±2.5	.003

Data are shown as the mean ± standard deviation or median [25th and 75th percentiles].

Abbreviations: HbA1c, glycated hemoglobin; SBP, systolic blood pressure; DBP, diastolic blood pressure.

[1] Comparison within groups: repeated measure one-way analysis of variance or Friedman's test.

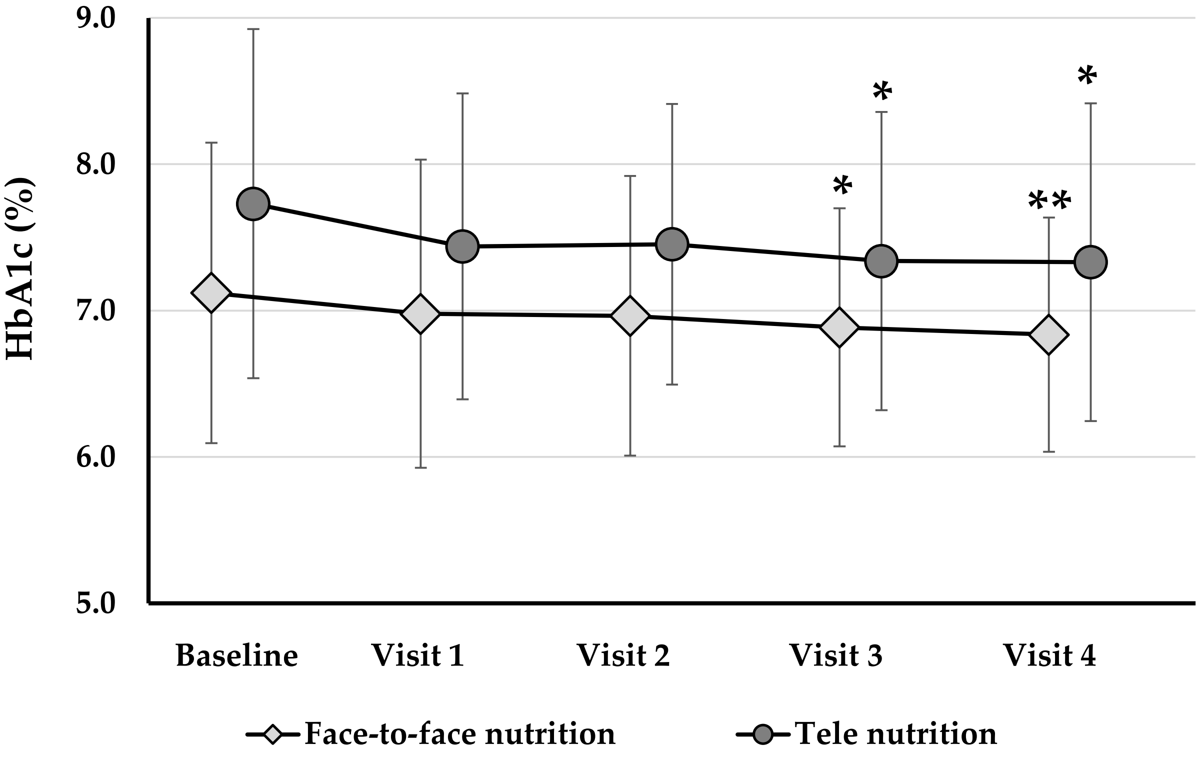

Figure S2. HbA1c change from baseline to the end of intervention for each group.

Data are shown as mean± standard deviation. Comparison within groups: repeated measure one-way analysis of variance, vs baseline; *p<0.05, **p<0.01.

CONSORT 2010 checklist of information to include when reporting a randomised trial*

Section/Topic	Item No	Checklist item	Reported on page No
**Title and abstract**			
	1a	Identification as a randomised trial in the title	1
	1b	Structured summary of trial design, methods, results, and conclusions (for specific guidance see CONSORT for abstracts)	1
**Introduction**			
Background and objectives	2a	Scientific background and explanation of rationale	1,2
	2b	Specific objectives or hypotheses	1,2
**Methods**			
Trial design	3a	Description of trial design (such as parallel, factorial) including allocation ratio	2
	3b	Important changes to methods after trial commencement (such as eligibility criteria), with reasons	None
Participants	4a	Eligibility criteria for participants	2
	4b	Settings and locations where the data were collected	2,4
Interventions	5	The interventions for each group with sufficient details to allow replication, including how and when they were actually administered	2-4
Outcomes	6a	Completely defined pre-specified primary and secondary outcome measures, including how and when they were assessed	4
	6b	Any changes to trial outcomes after the trial commenced, with reasons	None
Sample size	7a	How sample size was determined	5
	7b	When applicable, explanation of any interim analyses and stopping guidelines	None
Randomisation:			
Sequence generation	8a	Method used to generate the random allocation sequence	2
	8b	Type of randomisation; details of any restriction (such as blocking and block size)	2
Allocation concealment mechanism	9	Mechanism used to implement the random allocation sequence (such as sequentially numbered containers), describing any steps taken to conceal the sequence until interventions were assigned	2
Implementation	10	Who generated the random allocation sequence, who enrolled participants, and who assigned participants to interventions	2

Blinding	11a	If done, who was blinded after assignment to interventions (for example, participants, care providers, those assessing outcomes) and how	2,4
	11b	If relevant, description of the similarity of interventions	2-4
Statistical methods	12a	Statistical methods used to compare groups for primary and secondary outcomes	5
	12b	Methods for additional analyses, such as subgroup analyses and adjusted analyses	None
**Results**			
Participant flow (a diagram is strongly recommended)	13a	For each group, the numbers of participants who were randomly assigned, received intended treatment, and were analysed for the primary outcome	5,6
	13b	For each group, losses and exclusions after randomisation, together with reasons	5,6,Figure 2
Recruitment	14a	Dates defining the periods of recruitment and follow-up	2,4
	14b	Why the trial ended or was stopped	None
Baseline data	15	A table showing baseline demographic and clinical characteristics for each group	6,7, Table 1,
Numbers analysed	16	For each group, number of participants (denominator) included in each analysis and whether the analysis was by original assigned groups	5,6, Table 1
Outcomes and estimation	17a	For each primary and secondary outcome, results for each group, and the estimated effect size and its precision (such as 95% confidence interval)	7, 8, Table 2, Table 3, Figure 3
	17b	For binary outcomes, presentation of both absolute and relative effect sizes is recommended	7, 8, Figure 3
Ancillary analyses	18	Results of any other analyses performed, including subgroup analyses and adjusted analyses, distinguishing pre-specified from exploratory	None
Harms	19	All important harms or unintended effects in each group (for specific guidance see CONSORT for harms)	5,6
**Discussion**			
Limitations	20	Trial limitations, addressing sources of potential bias, imprecision, and, if relevant, multiplicity of analyses	10
Generalisability	21	Generalisability (external validity, applicability) of the trial findings	9-11
Interpretation	22	Interpretation consistent with results, balancing benefits and harms, and considering other relevant evidence	9-11
**Other information**			
Registration	23	Registration number and name of trial registry	2
Protocol	24	Where the full trial protocol can be accessed, if available	None
Funding	25	Sources of funding and other support (such as supply of drugs), role of funders	11

*We strongly recommend reading this statement in conjunction with the CONSORT 2010 Explanation and Elaboration for important clarifications on all the items. If relevant, we also recommend reading CONSORT extensions for cluster randomised trials, non-inferiority and equivalence trials, non-pharmacological treatments, herbal interventions, and pragmatic trials. Additional extensions are forthcoming: for those and for up to date references relevant to this checklist, see www.consort-statement.org.
